# A Case of Oral Lichen Planus Preceding the Diagnosis of Good Syndrome

**DOI:** 10.7759/cureus.64609

**Published:** 2024-07-15

**Authors:** Suzanne L Fastner, Jennie T Clarke

**Affiliations:** 1 Dermatology, Medical College of Wisconsin, Milwaukee, USA; 2 Dermatology, University of Utah School of Medicine, Salt Lake City, USA

**Keywords:** cyclosporine, pure red cell aplasia, common variable immunodeficiency, hypogammaglobinemia, thymoma, oral lichen planus, good syndrome

## Abstract

Good syndrome (GS) is a rare condition characterized by thymoma and immune deficiency with a poorly understood mechanism in which patients have reduced immunoglobulin levels and circulating B-cells along with impaired T-cell function. GS is often accompanied by autoimmune and inflammatory conditions, and in this report, we present a case of refractory oral lichen planus (OLP) preceding the diagnosis of GS. In this case, a patient with a history of OLP was diagnosed with GS and common variable immunodeficiency (CVID) following thymectomy and was treated with intravenous immunoglobin (IVIG). Additionally, he was found to have pure red cell aplasia managed with cyclosporine. His oral symptoms worsened, and he presented to dermatology. Treatment was initiated with topical clobetasol and tacrolimus for his OLP, and fluconazole was started for concomitant oral candidiasis. His OLP has remained under satisfactory control with this regimen; however, he requires close surveillance for malignancy given his increased risk of oral squamous cell carcinoma (OSCC) with immunosuppression and active OLP. Although rare, clinicians should be aware of GS and its association with erosive OLP along with the heightened risk of infection in these patients.

## Introduction

Good syndrome (GS) is a rare condition characterized by thymoma and hypogammaglobinemia with important mucocutaneous implications [1]. Herein, we present a case of GS associated with severe erosive oral lichen planus (OLP), a condition seen commonly in patients with GS.

## Case presentation

**Figure 1:**
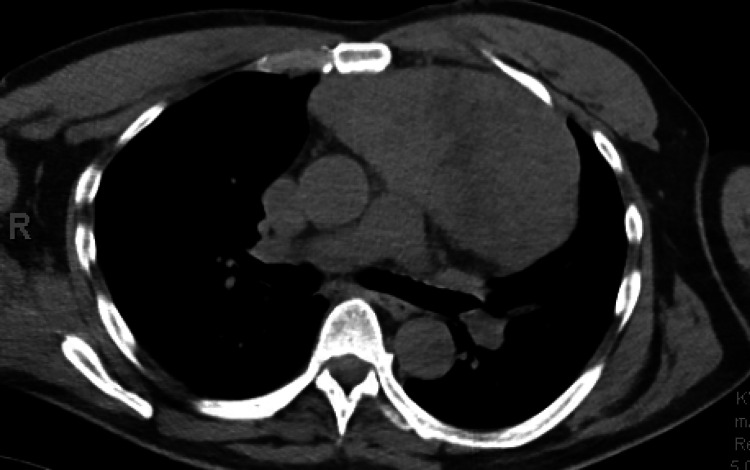
Chest CT showing an anterior mediastinal mass prior to thymectomy

In 2013, a middle-aged man with a history of OLP presented with a cough and fever and was found to have a 15.4 cm mediastinal mass on a chest radiograph. He underwent surgical removal of the mass, which was confirmed to be a thymoma (Figure 1). Additionally, at that time, he was also found to have low circulating immunoglobulin G, immunoglobulin A, and immunoglobulin M levels and was diagnosed with GS and common variable immunodeficiency (CVID) for which treatment with intravenous immunoglobin (IVIG) was initiated. The following year, he developed severe anemia requiring transfusions. Bone marrow biopsy identified pure red cell aplasia, and treatment with cyclosporine resolved his anemia.

**Figure 2:**
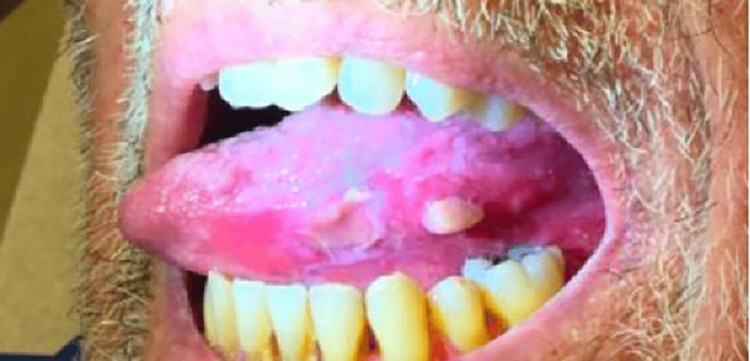
OLP of the left lateral tongue at the time of care establishment with dermatology nine years after GS diagnosis GS: good syndrome; OLP: oral lichen planus

**Figure 3:**
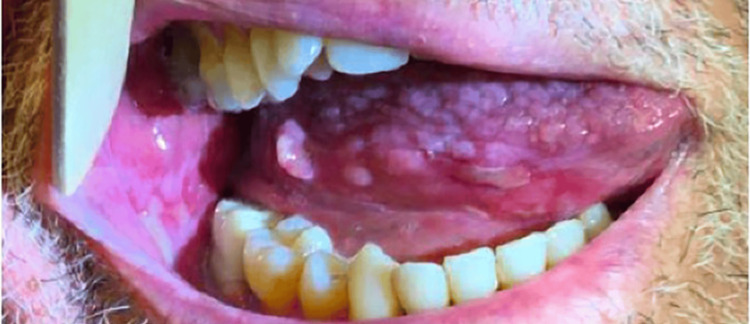
OLP of the right lateral tongue at the time of care establishment with dermatology nine years after GS diagnosis GS: good syndrome; OLP: oral lichen planus

**Figure 4:**
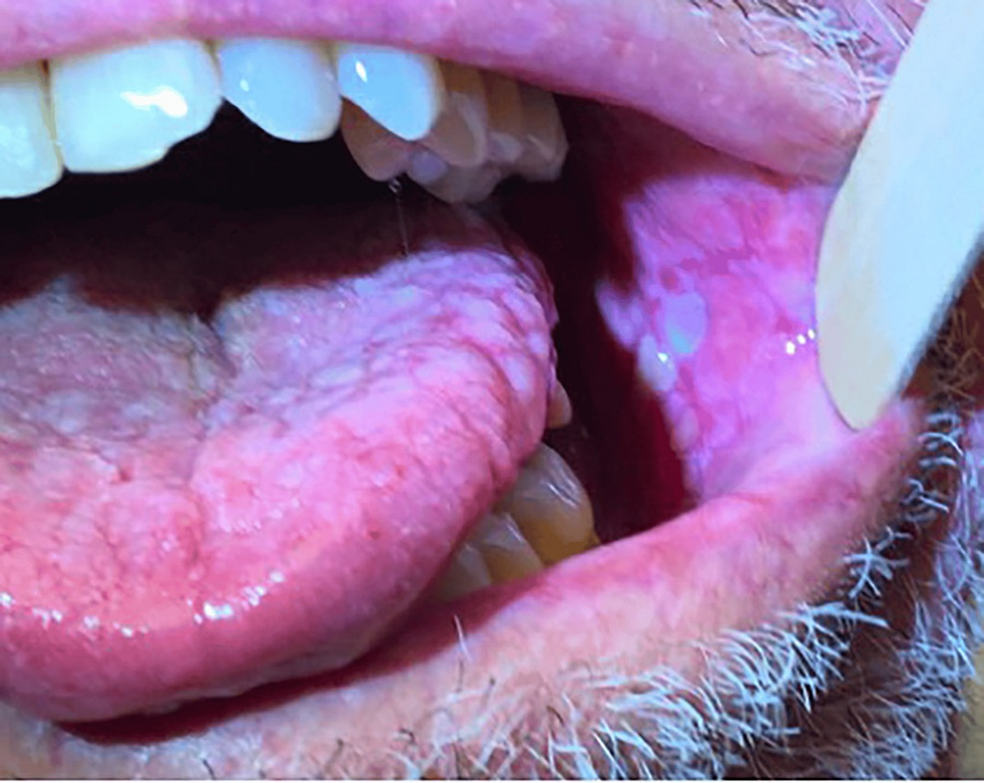
OLP of left inner cheek at the time of care establishment with dermatology nine years after GS diagnosis GS: good syndrome; OLP: oral lichen planus

A biopsy of an ulcer of the buccal mucosa was taken in 2016, confirming the patient’s OLP as evidenced by keratinocyte squamatization, scattered Civatte bodies, and a dense lichenoid infiltrate, flanked by an inflamed ulcer. At that time, the underlying submucosa demonstrated vascular proliferation and a dense mixed infiltrate of neutrophils, histiocytes, and eosinophils. Viral cytopathic changes were not identified in pathology. Several years later, he presented to dermatology for the management of OLP. He was having severe oral pain, erosions, and ulcerations along with gingival and lingual fibromas related to cyclosporine use (Figures 2, 3, 4). Treatment with clobetasol gel and tacrolimus “swish and spit” was initiated. He also intermittently required treatment with fluconazole for concomitant oral candidiasis. Since that time, he has been satisfied with the control of his OLP and declined additional systemic therapy. He also continues to receive IVIG and cyclosporine for the management of CVID and pure red cell aplasia. He is at increased risk of oral squamous cell carcinoma (OSCC) due to both his immunosuppression and OLP, and he remains under close surveillance for malignancy. 

## Discussion

GS is a rare condition characterized by thymoma and immune deficiency; patients have reduced immunoglobulin levels and circulating B-cells along with impaired T-cell function [2]. Proposed mechanisms for the association of thymoma and immunodeficiency include dysregulation of B- and T-cell growth cytokines or failure of B- and T-cell maturation [3]. GS is often accompanied by autoimmune and inflammatory conditions such as myasthenia gravis, pure red cell aplasia, and OLP, potentially due to T-cell dysregulation [2,4]. When present, OLP typically precedes thymoma identification. Some authors have included OLP as a clinical feature of GS, and some advocate screening for GS in patients with refractory erosive OLP (via chest radiograph and immunoglobulin levels) [3].

Patients with GS are also more susceptible to infections often due to pseudomonas bacteria, candida species, or cytomegalovirus, which are the leading causes of mortality for patients with GS [4]. While patients with GS almost always have low circulating immunoglobulins and B-cells, a study by Shi and Wang demonstrated no statistically significant difference in immunoglobulin levels or B-cell count for those who did develop humoral immunity-related infections compared to those who did not develop humoral immunity-related infections [4]. Additionally, the authors found no statistically significant difference in CD4 count or CD4/CD8 ratio for those who did develop cellular immunity-related infections compared to those who did not develop cellular immunity-related infections [4].

Thymectomy and IVIG are the mainstays of GS treatment [5]. Given the rarity of GS, studies evaluating the most effective treatment for GS-associated OLP are limited to small sample sizes. In patients with coexisting OLP and thymoma, there have been reports of improvement in OLP symptoms following thymectomy alone or thymectomy with IVIG administration, potentially explained by the autoimmune etiology of both GS and OLP; however, this response to thymectomy has not been consistent [3].

Although there have been very few reports describing cutaneous malignancy in GS patients, this patient will be closely monitored for OSCC [4,6]. Furthermore, he requires monitoring for infection, due to the high mortality associated with infection in GS. 

## Conclusions

Though rare, clinicians should be aware of GS and its association with erosive OLP and mucocutaneous infections. Infections in these patients should be treated aggressively. Given the association between GS, hypogammaglobinemia, and OLP, screening for GS with chest radiography and serum immunoglobulin levels could be considered for patients with recalcitrant OLP.

## References

[1] Ribatti D (2006). The fundamental contribution of Robert A. Good to the discovery of the crucial role of thymus in mammalian immunity. Immunol.

[2] Sipos F, Műzes G (2023). Good's syndrome: brief overview of an enigmatic immune deficiency. Acta Pathol Microbiol Scand.

[3] Le Gatt P, Nguyen AT, Baaroun V, Rochefort J (2023). Oral lichen planus in patients with Good’s syndrome: a literature review. Cureus.

[4] Shi Y, Wang C (2021). When the good syndrome goes bad: a systematic literature review. Front Immunol.

[5] Kelesidis T, Yang O (2010). Good's syndrome remains a mystery after 55 years: a systematic review of the scientific evidence. Clin Immunol.

[6] Jansen A, van Deuren M, Miller J (2016). Prognosis of Good syndrome: mortality and morbidity of thymoma associated immunodeficiency in perspective. Clin Immunol.

